# Clinical experience with misoprostol vaginal insert for induction of labor: a prospective clinical observational study

**DOI:** 10.1007/s00404-018-4942-y

**Published:** 2018-10-29

**Authors:** Markus Schmidt, Maria Neophytou, Olaf Hars, Julia Freudenberg, Maritta Kühnert

**Affiliations:** 1 Department of Gynecology and Obstetrics of the Sana Clinics Duisburg GmbH, Sanakliniken Duisburg, Zu Den Rehwiesen 3, 47055 Duisburg, Germany; 2 Beratung für Gute Wissenschaft, Goltzstr. 14, Berlin, Germany; 3 Department of Obstetrics and Perinatology, University Hospital of Marburg, Marburg, Germany

**Keywords:** Induction of labor, Misoprostol, Misoprostol vaginal insert, Mode of delivery, Prostaglandin E1, Time to delivery, Vaginal delivery

## Abstract

**Purpose:**

To provide real-world evidence using misoprostol vaginal insert (MVI) for induction of labor in nulliparous and parous women at two German Level I Centers in a prospective observational study.

**Methods:**

Between 1 August 2014 and 1 October 2015, eligible pregnant women (≥ 36 + 0 weeks of gestation) requiring labor induction were treated with MVI. Endpoints included time to and mode of delivery rates of tocolysis use, tachysystole, uterine hypertonus or uterine hyperstimulation syndrome and newborn outcomes.

**Results:**

Of the 354 women enrolled, 68.9% (244/354) achieved vaginal delivery (nulliparous, 139/232 [59.9%]; parous 105/122 [86.1%]; *p* < 0.001). Median time from MVI administration to vaginal delivery was 14.0 h (nulliparous, 14.5 h; parous, 11.9 h; *p* < 0.001). A total of 205/244 (84.0%) and 228/244 (93.4%) women achieved a vaginal delivery within 24 h and 30 h, respectively. The most common indications for cesarean delivery were pathologic cardiotocography (nulliparous, 41/232 [17.4%]; parous, 13/122 [10.7%]; *p* = 0.081) and arrested labor (dilation or descent; nulliparous, 45/232 [19.4%], parous, 3/122 [2.5%]; *p* ≤ 0.001). A total of 24.3% of women experienced uterine tachysystole and 9.6% experienced uterine tachysystole with fetal heart rate involvement, neither of which were significantly different for nulliparous and parous women. In total, 42/345 (12.2%) of the neonates had an arterial pH < 7.15 and 12/345 3.5% had a 5-min Apgar score ≤ 7.

**Conclusion:**

When clinically indicated, MVI was efficient and safe for induction of labor in women with an unfavorable cervix. Women, however, should be counseled regarding the risk of uterine tachysystole prior to labor induction with MVI.

## Introduction

Pregnancy is uncomplicated for the majority of women and progresses to term gestation and spontaneous labor without the need for intervention. In some cases, however, elective delivery by means of labor induction may be necessary due to maternal or fetal complications. Successful induction of labor is critically dependent upon the condition of the cervix. Onset of labor without prior cervical ripening occurs in approximately 5% of all pregnancies. This can cause the delivery to be protracted and complicated. When labor is induced, the condition of the cervix is particularly important. In the presence of an unripe cervix, a high rate of induction failures is experienced due to problems associated with poor cervical dilation. These include a prolonged and difficult course of labor, which frequently results in fetal distress, a high incidence of cesarean delivery, and other maternal and fetal complications, depending on the induction method used and parity [1]. Indeed, nulliparous women are more likely to have a long, unpredictable labor compared with parous women.

The importance of cervical ripeness has led to the development of a variety of techniques and treatments to prepare the cervix for induction of labor [2, 3] and thus to improve prognosis of delivery for both mother and fetus. In particular, misoprostol, an analogue of the naturally occurring prostaglandin E1 (PGE1), has been recognized as an agent for cervical ripening for many years [3, 4]. As a consequence, oral tablets of misoprostol have been used off-label for labor induction by clinicians since the 1990s [4].

Misoprostol vaginal insert (MVI; Misodel^®^/Mysodelle^®^/Myspess^®^, Ferring Pharmaceuticals) has been developed to provide continuous controlled-release of low-dose misoprostol, 7 µg/h over a period of 24 h [5], in an easy-to-use application for cervical ripening and labor induction, and is combined with an integral retrieval system to allow the insert to be easily and quickly removed at the end of the dosing period once onset of active labor is achieved or if an adverse event occurs. In many countries MVI is the only misoprostol product approved for induction of labor in women, using evidence from a large randomized controlled trial conducted in the United States [6, 7]. Compared with dinoprostone (prostaglandin E2, PGE2), misoprostol has shown enhanced uterine contractility effects [7–9], which offer advantages in terms of shorter time interval to delivery but also increases the risk for uterine tachysystole [7, 10, 11]. Shorter delivery time, however, was associated with reduced rates of maternal infection and antibiotic use [7] and may increase maternal satisfaction during the labor induction process [12]. When compared with the dinoprostone vaginal insert (DVI; Cervidil^®^/Propess^®^, Ferring Pharmaceuticals) MVI provided a shorter time to vaginal delivery and comparable overall incidence of maternal and neonatal adverse events [7].

Prior to the marketing authorization for MVI in Germany, the majority (two-thirds) of obstetricians who responded in a national survey stated that they used off-label misoprostol for labor induction for viable term pregnancies [13]. As such, despite concerns about potential legal consequences, many German obstetricians were familiar with the effectiveness and established clinical practice of labor induction with misoprostol tablet fragments [14]. Although MVI uses the same active compound as the off-label tablet formulations of misoprostol, it may decrease the time to vaginal delivery when compared with misoprostal tablet fragments, as well as possibly increasing the risk of uterine tachysystole in women who received MVI [15, 16]. As MVI is the only licensed formulation available for labor induction in Germany and many other countries it is important that its routine clinical use is well documented, particularly for time to delivery and any events that may affect maternal or neonatal outcomes, such as uterine tachysystole with or without fetal heart rate (FHR) involvement.

The aim of the present prospective clinical observational study was to systematically document German daily clinical routine experience with MVI in a wide range of indications for labor induction, including women with premature rupture of membranes. Our objectives were to determine by parity how many women induced with MVI delivered within 24 h, the proportion of women with a failed induction (no delivery within 30 h), how many had a vaginal, operative vaginal or cesarean delivery, and median time from MVI administration to onset of adverse events (including uterine hypertonus, uterine tachysystole, uterine hyperstimulation syndrome, a non-reassuring fetal heart rate [FHR] pattern defined as International Federation of Gynecology and Obstetrics [FIGO] score < 8, and need for tocolysis).

## Materials and methods

This prospective unilateral clinical observational study was performed between 1 August 2014 and 1 October 2015 in two perinatal Level I centers in Germany (Department of Obstetrics and Perinatology of the University Hospital of Marburg and Department of Gynecology and Obstetrics of the Sana Clinics Duisburg GmbH). The study was approved by the relevant Institutional Review Board and the local ethical committees of both clinics. Informed consent was obtained from all individual participants included in the study. The data of 354 consecutive nulliparous and parous pregnant women aged ≥ 18 years in whom labor was induced with MVI were pooled and analyzed.

Women with either a maternal or fetal medical indication for labor induction were treated with MVI if the following criteria were met: 37 weeks of gestation (36 + 0), parity < 3, singleton pregnancy, body mass index (BMI) < 50 kg/m^2^ and unfavorable cervix (Bishop Score < 4). Indications for induction of labor were: exceeding term dates (> 40 + 0 weeks of gestation), premature rupture of membranes, diabetes or gestation diabetes, fetal growth retardation, pregnancy-induced. Women were not eligible for treatment with MVI if hypersensitive to the active substance or to any of the excipients of MVI, labor had already started, there was suspicion or evidence of fetal compromise prior to induction, they had received oxytocic drugs and/or other labor induction agents, there was suspicion or evidence of uterine scars resulting from previous uterine or cervical surgery (e.g., cesarean delivery), there was uterine abnormality (e.g., malformations), there was placenta previa or any other contraindication for attempted vaginal delivery, there was unexplained vaginal bleeding after 24 weeks of gestation, there was fetal malpresentation, or there were signs or symptoms of chorioamnionitis unless adequate prior treatment has been administered. In addition, pregnant women were excluded from treatment with the MVI if they had severe pre-eclampsia marked by hemolytic anemia, elevated liver enzymes, low platelet count (HELLP syndrome), other end-organ affliction or central nervous system findings other than mild headache. Women with evidence of pre-eclampsia or with a suspicion of fetal compromise were excluded from the study prior to treatment with MVI. Group B *Streptococcus* positive women were offered intravenous antibiotics prophylaxis prior to labor induction with MVI, which was repeated every 4 h until delivery in order to achieve adequate protection against neonatal *Streptococcus* infections.

Demographic data and baseline characteristics were recorded: maternal age, BMI, parity, modified Bishop score, membrane status, gestational age at the time of MVI placement, and the indication for induction of labor.

The pregnant women received one MVI (200 µg misoprostol, controlled-release of 7 µg/h over 24 h) placed in the posterior vaginal fornix. If required a water-soluble gel was used to aid correct positioning of the insert. In women with premature rupture of membranes (PROM), labor was induced if there were no contractions within 12 h but less than 24 h had passed since the occurrence of PROM. Intravenous antibiotics were started 12 h after PROM.

The vaginal insert was removed when active labor was achieved (defined as three or more contractions within 10 min, lasting 45 s or longer, and which resulted in cervical change OR a cervical dilation of at least 4 cm with any frequency of contractions), or after completion of the 24-h dosage period, as reported by Wing et al. [7]. Generally, there was an interval of at least 30 min between the removal of the vaginal insert and the start of intravenous pre-delivery oxytocin administration, if necessary, according to the summary of product characteristics [5].

Each patient underwent 30 min of cardiotocography (CTG) assessment before and 60 min after insertion of MVI to record the fetal status and to confirm that there was no active labor or fetal distress. CTG assessments were performed every 3–4 h and permanently during active labor, following PROM or if there was any bleeding.

If required, tocolysis with fenoterol as a bolus, or if this did not suffice as an infusion, was performed depending on the maternal and fetal conditions in women with uterine tachysystole with or without pathologic CTG, defined as any Category II or III FHR pattern.

The primary endpoints of time to and mode of delivery (vaginal, cesarean, operative vaginal) were recorded. Additional endpoints were rates of vaginal and any delivery within 24 and 30 h, time from placement of MVI to vaginal delivery, cesarean delivery, onset of active labor and MVI removal, total time in delivery room, rate of cesarean deliveries and indications, rate of operative vaginal deliveries, proportion of emergency cesarean deliveries, proportion of women requiring pre-delivery oxytocin, rate of tocolysis to treat FHR abnormalities, uterine tachysystole, uterine hypertonus or uterine hyperstimulation syndrome. The proportion of women who received epidural, intravenous or other type of analgesia was also recorded. All endpoints were assessed by parity.

The rates of pathologic CTG with any Category II/III FHR pattern, uterine tachysystole, uterine hypertonus, or uterine hyperstimulation syndrome were also calculated. Uterine tachysystole was defined as five or more contractions within 10 min, averaged over three consecutive 10-min periods. Uterine hypertonus was defined as increased basal tonus of the uterus or a uterine contraction lasting more that 2 min as assessed using CTG monitoring. Uterine hyperstimulation syndrome included uterine tachysystole, uterine hypertonus and a pathological FHR pattern. Fetal outcome was benchmarked by the proportion of neonates with 5-min Apgar Score ≤ 7, and an umbilical artery pH < 7.15.

Failed induction was also analyzed using the definition of MacVicar [17] which included all women for whom the uterus failed to contract after adequate stimulation or amniotomy, or the uterus contracted abnormally and the cervix did not dilate completely. In this study failed induction was defined when there was no delivery > 30 h after the start of induction. Women were followed up for 2 h in the delivery unit after vaginal and operative vaginal delivery, and for 6 h after cesarean delivery.

All patients who met the inclusion criteria between 1 August 2014 and 1 October 2015 were consecutively enrolled into the study. As such, there was no formal calculation for the size population size. Statistical Package for Social Sciences (SPSS, IBM Version 22) was used to conduct statistical analysis of the data, which included the calculation of mean, median and standard deviation (SD) and confidence interval (CI) values. The level of statistical significance was set at 0.05. The skewness and kurtosis of the distribution for all variables were evaluated using the Kolmogorov–Smirnov test for normal distribution. The Mann–Whitney *U* test was used to assess differences for variables that were not normally distributed. The Wilcoxon test was used to assess differences in measurement cycle dependent variables. The Chi-squared and Fisher’s exact test was applied in frequency comparisons.

## Results

A total of 354 women were included in the study; 232 (65.5%) women were nulliparous and 122 (34.5%) women were parous. Demographic parameters are presented in Table 1. No relevant differences were observed between nulliparous and parous women. The majority of women delivered within 30 h of the start of induction: 207/232 (89.2%) nulliparous women and 114/122 (93.4%) parous women. A total of 60% of women received epidural anesthesia, 20% received intravenous analgesia, and 5% received other types of analgesia, including suppositories.

**Table 1:** Demographic parameters

Parameter	Nulliparous (*n* = 232)	Parous (*n* = 122)	Total (*n* = 354)	*p* value*
Maternal age, (years) (mean ± SD)	29.0 ± 5.8	32.7 ± 5.2	30.3 ± 5.9	≤ 0.001
Gestational age, weeks median (95% CI)	41.0 (40–41)	40.0 (39–40)	40.0 (40–41)	0.001
BMI, (kg/m^2^) (mean ± SD)	26.7 ± 6.6	27.8 ± 6.9	27.1 ± 6.7	0.137
Membrane intact at MVI placement, *n* (%)	196 (84.5)	99 (81.1)	295 (83.3)	0.363

*CI* confidence interval

*Chi-squared test was used for nominal data (%) and Mann–Whitney *U* test was used for nonparametric variables (median)

The most common indications for labor induction in both nulliparous and parous women were exceeding term date defined as > 40 + 0 weeks of gestation (38.1%), PROM (16.7%), diabetes or gestational diabetes (8.8%), and fetal growth retardation (6.2%) (Table 2). There were no statistical differences between nulliparous and parous women for indications for labor induction.

**Table 2:** Indications for induction of labor

Indication	Nulliparous (*n* = 232)		Parous (*n* = 122)		Total (*n* = 354)		*p* value*
	*n*	%	*n*	%	*n*	%	
Exceeding term date (> 40 + 0 weeks of gestation)	95	40.9	40	32.8	135	38.1	0.133
Premature rupture of membranes	36	15.5	23	18.9	59	16.7	0.424
Diabetes/gestational diabetes	16	6.9	15	12.3	31	8.8	0.068
Fetal growth retardation	18	7.8	4	3.3	22	6.2	0.097
Pregnancy-induced hypertonia	11	4.7	6	4.9	17	4.8	0.941
Fetal macrosomia	8	3.4	6	4.9	14	4.0	0.500
Pre-eclampsia	7	3.0	5	4.1	12	3.4	0.593
Other	41	17.7	23	18.9	64	18.1	0.784

*Chi-squared test was used for nominal data (%)

**Fig. 1:**
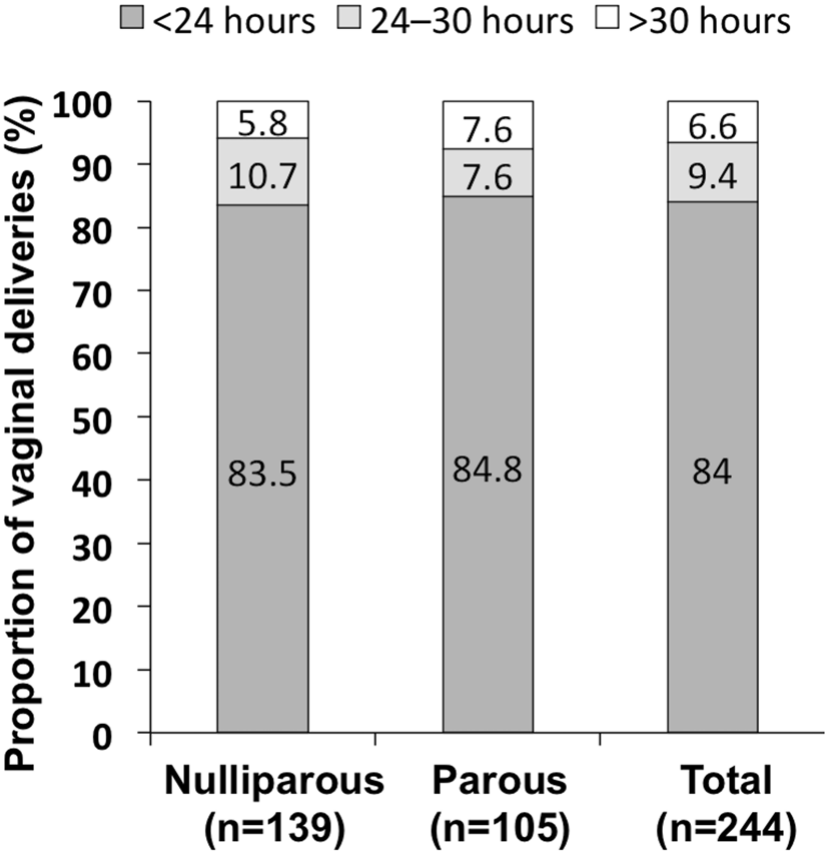
Time to vaginal delivery

Delivery outcome parameters are presented in Table 3. A successful vaginal delivery following induction with MVI occurred in 68.9% (*n* = 244) with a significant difference between the nulliparous and parous group (59.9% vs 86.1%, *p* < 0.001). The median time from administration of the MVI to vaginal delivery for women of any parity was 14.0 h, with a significant difference between nulliparous and parous women (14.5 h vs 11.9 h, *p* = 0.014). Of the 244 women who had vaginal deliveries 84.0% occurred within 24 h and 93.4% within 30 h (no significant difference between nulliparous and parous women; Fig. 1).

**Table 3:** Delivery-related outcomes

Outcome parameter	Nulliparous	Parous	Total	*p* value*
Vaginal delivery,* n*/N (%)	139/232 (59.9)	105/122 (86.1)	244/354 (68.9)	< 0.001
Vaginal delivery within 12 h,* n*/N (%)	40/139 (28.8)	53/105/ (50.5)	93/244/ (38.1)	0.001
Vaginal delivery within 24 h,* n*/N (%)	116/139 (83.5)	89/105 (84.8)	205/244 (84.0)	n.s.
Vaginal delivery within 30 h,* n*/N (%)	131/139 (94.2)	97/105 (92.4)	228/244 (93.4)	n.s.
Time to vaginal delivery, h median (95% CI)	14.5 (13.6–16.1)	11.9 (10.6–14.0)	14.0 (13.1–14.8)	0.014
Time to insert removal, h median (95% CI)	9.5 (1.5–29.0)	8.9 (1.3–24.0)	9.2 (1.3–29.0)	n.s.
Time to onset of active labor, h median, (95% CI)	4.2 (3.2–5.3)	2.2 (1.6–2.8)	3.2 (2.9–3.8)	< 0.001
Any delivery within 12 h,* n*/N (%)	67/232 (28.9)	63/122 (51.6)	130/354 (36.7)	< 0.001
Any delivery within 24 h,* n*/N (%)	178/232 (76.7)	105/122 (86.1)	283/354 (79.9)	0.037
Any delivery within 30 h*, n/N* (%)	207/232 (89.2)	114/122 (93.4)	321/354 (90.7)	n.s.
Time to any delivery, h median (95% CI)	14.9 (14.2–16.2)	11.8 (10.3–13.4)	14.3 (13.4–14.9)	< 0.001
Women requiring pre-delivery oxytocin, *n/N* (%)	20/232 (8.6)	2/122 (1.6)	22/354 (6.2)	< 0.001
Women requiring tocolysis,* n*/N (%)	43/232 (18.5)	14/122 (11.5)	57/354 (16.1)	0.014
Cesarean delivery,* n*/N (%)	93/232 (40.1)	17/122 (13.9)	110/354 (31.1)	< 0.001
Emergency cesarean delivery,* n*/N (%)	7/232 (3.0)	5/122 (4.1)	12/354 (3.4)	n.s.
Operative vaginal delivery, *n* (%)	28/232 (12.1)	1/122 (0.8)	29/354 (8.2)	< 0.001
Time in delivery room, h median (95% CI)	5.6 (0.03–32.4)	3.2 (0.1–12.7)	4.7 (0.03–32.4)	< 0.001

*CI* confidence interval

*Chi-squared test was used for nominal data (%) and Mann–Whitney *U* test was used for nonparametric variables (median)

The median time from administration of MVI to any delivery was 14.3 h, with a significant difference between nulliparous and parous women (14.9 h vs 11.8 h, *p* < 0.001). Overall, in 79.9% of the women any delivery occurred within 24 h and in 90.7% within 30 h.

The overall cesarean delivery rate was 31.1% (110/354) with a significantly higher incidence in nulliparous than parous women (40.1% vs 13.9%, *p* < 0.001). The most common indications for a cesarean delivery were pathologic CTG with Category II/III FRH pattern (16.7% [54/354]) and arrested labor with failure of birth progress dilatation or failure to dilate (total 15.6% [48/354]; 19.4% for nulliparous women vs 2.5% for parous women; *p* ≤ 0.001; Table 4). The overall rate of emergency cesarean deliveries for the total nulliparous and parous women regardless of delivery method was similar (3.0% and 4.1%, respectively, not significantly different). Vaginal operative deliveries were performed in 8.2% of all women with a significantly higher proportion in nulliparous than parous women (12.1% [28/232] vs 0.8% [1/122], *p* < 0.001).

**Table 4:** Indications for cesarean delivery

Reasons *n* (%)	Nulliparous (*n* = 232)	Parous (*n* = 122)	Total (*n* = 354)	*p* value*
Pathologic CTG	41/232 (17.6)	13/122 (10.7)	54/354 (16.7)	0.081
Arrested labor (dilation or descent)	45/232 (19.4)	3/122 (2.5)	48/354 (15.6)	≤ 0.001
Maternal reasons	4/232 (1.7)	0/122 (0)	4/354 (1.1)	n.s.
Chorioamnionitis	1/232 (0.4)	0/122 (0)	1/354 (0.3)	n.s.
Not known	2/232 (0.9)	1/122 (0.8)	3/354 (0.8)	n.s.

*Chi-squared test was used for nominal data (%)

*n.s.* not significant

The mean arterial and venous pH values were 7.24 and 7.31, respectively. In total, 42/345 (12.2%) of the neonates had an arterial pH < 7.15 with a significantly higher incidence in the nulliparous group (34/229 [14.8%] vs 8/116 [6.9%], *p* = 0.036). There were no cases with an arterial pH < 7.0. There were 12/345 (3.5%) neonates who had a 5-min Apgar score ≤ 7 (Table 5). Two neonates were admitted to the Neonatal Intensive Care Unit, both born to parous women with pregnancies more than 40 + 0 weeks of gestation. One neonate had polycystic kidneys, the other was admitted for observation because of adaption disorder plus an arterial pH of 7.07 at birth caused by maternal blood pressure problems during epidural anesthesia, and had a quick recovery.

**Table 5:** Neonatal safety outcomes

Outcome	Nulliparous (*n* = 229)	Parous (*n* = 116)	Total (*n* = 345)	*p* value*
Arterial pH < 7.15,* n*/N (%)	34/229 (14.8)	8/116 (6.9)	42/345 (12.2)	0.036
5-min Apgar score ≤ 7,* n*/N (%)	9/229 (3.9)	3/116 (2.6)	12/345 (3.5)	n.s.

Missing data for neonates delivered by three nulliparous and six parous women

*n.s.* not significant

*Chi-squared test was used for nominal data (%)

Important safety outcomes are presented in Table 6. Transient pathologic CTG with any Category II/III FHR pattern was recorded for 35.0% of the women and uterine tachysystole in 24.3%. The occurrence of uterine tachysystole plus FHR abnormality occurred in 9.6% of the women. No uterine hyperstimulation syndrome or uterine hypertonus were observed. Atony with a postpartum hemorrhage was reported in three women (0.8%) and retained placenta in one woman (0.3%). There were no fetal, maternal, or neonatal deaths.

**Table 6:** Important fetal and maternal safety outcomes

Reasons *n*/N (%)	Nulliparous (*n* = 232)	Parous (*n* = 122)	Total (*n* = 354)	*p* value*
FHR abnormality	97/232 (41.8)	27/122 (22.1)	124/354 (35.0)	< 0.001
Uterine tachysystole	57/232 (24.6)	29/122 (23.8)	86/354 (24.3)	n.s.
Tachysystole + FHR abnormality	24/232 (10.3)	10/122 (8.2)	34/354 (9.6)	n.s.
Postpartum atony	1/232 (0.4)	2/122 (1.6)	3/354 (0.8)	n.s.
Placenta retention	1/232 (0.4)	0/122 (0.0)	1/354 (0.3)	n.s.

Multiple occurrences possible. FHR, fetal heart rate; no occurrences of uterine hypertonus or uterine hyperstimulation syndrome were reported in our study. Uterine hyperstimulation syndrome included uterine tachysystole or hypertonus AND a pathological FHR pattern

*n.s.* not significant

*Chi-squared test for nominal data (%)

MVI was removed after a median time of 9.2 h. Women stayed in the delivery room for a median of 4.7 h until delivery. The median duration in the delivery room was significantly longer for nulliparous women compared with parous women (5.6 h vs 3.2 h, *p* < 0.001). A total of 6.2% of women required oxytocin. Intrapartum tocolysis was required in 16.1% of women in the study. Failure of induction was seen in 9.3% of the study population, defined as no delivery within 30 h.

## Discussion

This prospective clinical observational study confirmed the efficacy of MVI (controlled and sustained-release misoprostol 7 µg/h for up to 24 h) in both nulliparous and parous women who all had medical reasons for induction of labor, such as being post-term (> 40 + 0 weeks of gestation) diabetes, or hypertension. In our study, the median time from administration of the MVI to vaginal delivery for women of any parity was 14.0 h. This is comparable with the lately published data of Mayer et al. [18] and Jagielska et al. [19], but lower than in the phase 3 EXPEDITE trial by Wing et al. [7] who reported a median time to vaginal delivery of 21.5 h. Another study by Bolla et al. [16] that included 200 women who received MVI had a mean time from start of induction to vaginal delivery of approximately 17 h and 45 min. In our study, the parous group had a median time to vaginal delivery of 11.9 h, which is comparable with data published by Mayer et al. [18] and the EXPEDITE trial by Wing et al. [7]. The difference in median time to vaginal delivery may be explained by a higher rate of women with PROM of 16.7% in our study versus 3.7% in the EXPEDITE trial [7]. A subgroup analyses of our patients with PROM showed that the median time to vaginal delivery was even shorter with 12.5 h in the nulliparous and 8.8 h in the parous group. Furthermore, the study population of the EXPEDITE trial by Wing et al. included women of different ethnicities and a higher mean BMI. Many other factors also affect time to delivery, including baseline cervical ripeness and use of epidural anesthetic.

The decreased time to vaginal delivery observed with MVI has several benefits, including a reduced need of antibiotics and pre-delivery oxytocin. In our study, as the median stay in the delivery room was below 5 h, which indicates reduced utilization of hospital resources, namely personnel staff, and allows induction of labor procedures to be planned according to staff rotas and the number of delivery suites available.

The cesarean delivery rate of 31.1% in our study was slightly higher than in the phase 3 EXPEDITE trial in which 26% of women who received MVI had a cesarean delivery [7]. Other studies have reported various rates of cesarean deliveries after induction with MVI, including a retrospective cohort study reported by Mayer et al. (10.1%) [18] and a pair-matched case–control study by Döbert et al. (39.1%) [6]. The reasons for the different cesarean delivery rates among the different studies remain unclear and comparisons between different studies should be cautious due to different patient populations. One explanation for the differences in cesarean delivery rates could be that the women induced with MVI in the various studies had different underlying risk factors and medical complications. For example, approximately 10% of women who took part in the EXPEDITE trial had elective inductions [20], whereas in our study, all the women had medical reasons for labor induction. Furthermore, women with medical reasons for induction may have other confounding factors that influence the labor process, for example, hypertensive disorders are often more common in women with higher BMI. As such, an accumulation of factors, including gestational age, maternal age, BMI, baseline mBS, and epidural analgesia in combination with medical complications, all will impact the likelihood of whether a women requires a cesarean delivery. Nevertheless, the range in cesarean delivery rate after labor induction with MVI is similar to a population-based study that included 42,950 births in Victoria, Australia, between 2000 and 2015, in which 26.5% of nulliparous women who had labor induction had a cesarean delivery (the induction methods included amniotomy, pre-delivery oxytocin and/or prostaglandins) [21].

In our study, the two most frequent reasons for cesarean delivery were a pathologic CTG with a Category II/III FHR pattern for parous women, and arrested labor (dilation or descent) in nulliparous women. As such, although parous women were significantly less likely to have a cesarean delivery when induced with MVI, among parous women who did have a cesarean delivery, the rate of emergency cesareans was higher than for nulliparous women who had a cesarean delivery. This can be explained by the different indications for cesareans by parity (i.e., arrest of labor does not usually result in emergency cesareans, whereas an emergency cesarean is more likely with Category II/III FHR patterns).

One well-known adverse effect of prostaglandins used for labor induction is uterine tachysystole and uterine hyperstimulation syndrome [22, 23]. In our study, uterine tachysystole was reported in 24.3% women and 9.6% of women had both uterine tachysystole and FHR abnormalities. Tocolysis was required in 16.1% of women. Uterine hyperstimulation syndrome or uterine hypertonus were not seen in our study. In the EXPEDITE trial, however, 49.1% of women in the MVI group experienced ‘Any tachysystole’ (including both adverse events and non-adverse events) [7].

It is important to evaluate neonatal outcomes to assess the benefits of reduced time to delivery and reduction of protracted labor against the incidence of tachysystole occurring with the MVI. Despite the occurrence of tachysystole and pathological CTGs the overall fetal outcome of the neonates was good. 87.8% of the neonates had an umbilical arterial pH value ≥ 7.15 and 96.5% had a 5-min Apgar score ≥ 7. Although there were more neonates with an arterial pH value < 7.15 born to nulliparous women compared with parous women, this is likely to be due to nulliparous women having a longer average duration of labor, including second stage rather than other risk factors for fetal acidosis. Importantly, no neonates in our study had an arterial pH value < 7.0, below which is associated with neonatal morbidity [24].

Induction of labor has become more common during the last decade. In developed countries, up to 25% of all deliveries at term now involve induction of labor [25]. As induction of labor should only be performed if there is a clear medical indication, taking into account expected benefits and potential harms, the method of induction of labor should be efficient, save, easy to handle and well tolerated with few adverse effects for the parturient and the fetus. Our observational study enhances our understanding of using MVI for induction of labor in general clinical practice, particularly because we enrolled consecutive women who had labor induced with MVI, thus limiting potential bias in patient selection. Furthermore, the inclusion criteria meant that a wide range of women were eligible for labor induction with MVI. Nevertheless, due to the observational nature of our study, the results should be interpreted cautiously as there is no control or comparison group. The results of our study provide real-world evidence of using MVI in general clinical practice, showing that the majority of women achieve delivery within 30 h of the start of induction; this is particularly relevant as these women had medical reasons why continuing pregnancy was potentially unsafe for either the mother or fetus. Our study also highlights that women who are induced with MVI should be carefully monitored in case there are maternal AEs or FHR abnormalities that require management.

In summary, our observational study supports the use of MVI for induction of labor and delivery of neonates within a relatively short time frame. When there is an existing medical indication for induction of labor the short duration of labor can be considered the main benefit of MVI. Furthermore, MVI has been approved in Europe and many other countries for induction of labor. MVI is recommended by the Swiss Society of Gynecology and Obstetrics as the prostaglandin of choice for IOL in the approved indications [26].

Before using MVI, women should be counseled about the risk of uterine tachysystole, which might require acute or prolonged tocolysis. Overall, the MVI was efficient and safe for induction of labor in women with an unfavorable cervix.
